# Human Immunodeficiency Virus Type-1 Elite Controllers Maintain Low Co-Expression of Inhibitory Receptors on CD4+ T Cells

**DOI:** 10.3389/fimmu.2018.00019

**Published:** 2018-01-22

**Authors:** Kajsa Noyan, Son Nguyen, Michael R. Betts, Anders Sönnerborg, Marcus Buggert

**Affiliations:** ^1^Division of Clinical Microbiology, Department of Laboratory Medicine, Karolinska Institutet, Stockholm, Sweden; ^2^Department of Microbiology, Perelman School of Medicine, University of Pennsylvania, Philadelphia, PA, United States; ^3^Division of Infectious Diseases, Department of Medicine Huddinge, Karolinska Institutet, Stockholm, Sweden; ^4^Center for Infectious Medicine, Department of Medicine Huddinge, Karolinska Institutet, Stockholm, Sweden

**Keywords:** human immunodeficiency virus type-1, elite controllers, CD4+ T cells, T cell exhaustion, inhibitory receptors, programmed cell death-1, CTLA-4, TIGIT

## Abstract

Human immunodeficiency virus type-1 (HIV-1) elite controllers (ELCs) represent a unique population that control viral replication in the absence of antiretroviral therapy (cART). It is well established that expression of multiple inhibitory receptors on CD8+ T cells is associated with HIV-1 disease progression. However, whether reduced co-expression of inhibitory receptors on CD4+ T cells is linked to natural viral control and slow HIV-1 disease progression remains undefined. Here, we report on the expression pattern of numerous measurable inhibitory receptors, associated with T cell exhaustion (programmed cell death-1, CTLA-4, and TIGIT), on different CD4+ T cell memory populations in ELCs and HIV-infected subjects with or without long-term cART. We found that the co-expression pattern of inhibitory receptors was significantly reduced in ELCs compared with HIV-1 cART-treated and viremic subjects, and similar to healthy controls. Markers associated with T cell exhaustion varied among different memory CD4+ T cell subsets and highest levels were found mainly on transitional memory T cells. CD4+ T cells co-expressing all inhibitory markers were positively correlated to T cell activation (CD38+ HLA-DR+) as well as the transcription factors Helios and FoxP3. Finally, clinical parameters such as CD4 count, HIV-1 viral load, and the CD4/CD8 ratio all showed significant associations with CD4+ T cell exhaustion. We demonstrate that ELCs are able to maintain lower levels of CD4+ T cell exhaustion despite years of ongoing viral replication compared with successfully cART-treated subjects. Our findings suggest that ELCs harbor a “healthy” state of inhibitory receptor expression on CD4+ T cells that might play part in maintenance of their control status.

## Introduction

Effective T cell responses are essential for control of pathogens and cancer. During human immunodeficiency virus type-1 (HIV-1) infection, constant viral replication and persistent exposure of antigen to virus-specific T cells leads to failure of cellular immune responses to fully launch an effective response and eradicate the virus. As a consequence, CD4+ T cells are progressively lost, leading to the development of AIDS without combined antiretroviral therapy (cART) (1). However, a very small subset of HIV-infected individuals, termed HIV elite controllers (ELCs), are able to spontaneously control viral replication to almost undetectable levels for many years without cART (2–4). An improved understanding of the factors associated with the natural control of viral replication in ELCs is essential and would provide vital information relevant for HIV-1 eradication and functional cure strategies.

The persistent exposure of antigens drives virus-specific T cells to an altered transcriptional state where they become dysfunctional (5). This phenomenon has been known as T cell exhaustion and is characterized by poor effector functions and high expression of several inhibitory receptors following chronic antigen exposure (6). The upregulation of multiple inhibitory receptors cooperates to limit T cell activation, thereby impeding fully functional immunity (7). Several studies have identified numerous inhibitory receptors that are elevated on T cell in several tumors and HIV-1 infection; such as programmed cell death-1 (PD-1), cytotoxic T-lymphocyte associated protein 4 (CTLA-4 and CD152), CD244 (2B4), CD160, lymphocyte activation gene 3 (LAG-3), T cell immunoglobulin and mucin 3 (Tim-3), and T-cell immunoglobulin and ITIM domain (TIGIT), among others (8–15). Importantly, blockade of these inhibitory receptors improves functional responses of T cells and has changed the landscape of anticancer therapies (16, 17). As such, it remains important to understand to what degree T cell exhaustion play in immunopathogenesis and natural control of chronic viral diseases, including HIV-1.

During recent years, extensive research has been made to define the molecular pathways and transcriptional pathways involved in exhausted versus memory CD8+ T cells (18, 19). Despite the extensive knowledge of CD8+ T cell exhaustion in mice and humans, much less is known about exhaustion in the setting of CD4+ T cells. Recent studies in mice have demonstrated a relationship between the transcription factors FoxP3 and Ikzf2 (Helios), and an exhausted state for CD4+ T cells in chronic LCMV infection (20), findings that could be of translational importance in HIV-1 disease. In the setting of HIV-1, studies have shown that HIV-infected subjects harbor increased levels of CD4+ T cells expressing PD-1, Lag-3, CTLA-4, and 2B4, among others (11, 12, 21–23). Furthermore, several studies on ELCs have shown that natural control of HIV-1 is associated with more vigorous and polyfunctional responses of HIV-specific CD4+ T cells (24, 25). However, at this point, it remains unknown whether low levels of multiple inhibitory receptors on memory CD4+ T cells is linked to spontaneous HIV-1 control and altered expression of exhaustion-related transcription factors; findings that could lead to insight into future directives of effective vaccine and cure strategies.

In this study, we examined the expression of multiple inhibitory receptors on memory CD4+ T cells in ELCs compared with other HIV-1-infected subjects and aimed to define if CD4+ T cell exhaustion is more prominent in certain CD4+ T cell subsets than others. We provide evidence that ELCs have lower expression of multiple measurable inhibitory receptors on CD4+ T cells in comparison to other HIV-infected individuals and that ELCs are able to maintain a “healthy” state of CD4+ T cell exhaustion despite ongoing low levels of chronic antigen exposure for many years.

## Materials and Methods

### Study Participants and Cell Preparations

An observational cohort of 46 HIV-1-infected individuals was recruited to the study. Study participants were divided into three groups: (1) ELCs were recruited from clinics around Sweden, these included the HIV Outpatient Clinic at Karolinska University Hospital Huddinge and Stockholm South General Hospital (Stockholm, Sweden); Infection Clinic Västmanlands Hospital (Västerås, Sweden); and Infection Clinic Gävle Hospital (Gävle, Sweden). All Swedish HIV-1-infected patients were evaluated with regard to established criteria for ELCs using the national InfCare HIV database, and 22 patients were identified. Of those 19 patients approved to participate in the study and met either of the two following criteria; HIV-1 positive status for minimum 1 year with HIV-1 RNA levels below 75 copies/ml on minimum three consecutive determinations, spanning over at least 12-month cART-free period with all previous determinations below 1,000 copies/ml (*n* = 15) (26); or HIV-1 positive status for ≥10 years with minimum two HIV-1 RNA level determinations with ≥90% of all HIV-1 RNA determinations below 400 copies/ml (*n* = 4) (27). (2) Untreated viremic patients (viremic) (*n* = 8). (3) cART treated (cART+): 19 patients with long-term usage of cART, between 13 and 24 years, and successful viral suppression below detection limit during this time period. The latter two groups were derived from Karolinska University Hospital. In addition, an age- and sex-matched healthy control (HC) group of 17 HIV-1 seronegative individuals was recruited to the cohort.

The demographic and clinical characteristics of all study subjects are summarized in Table 1. The ELC patient group displayed a significant higher median (IQR) CD4+ T cell count [950 cells/mm^3^ (695–1,655)] than subjects on long-term cART [550 cells/mm^3^ (490–610)]. The detailed patient characteristics of ELCs are given in Table S1 in Supplementary Material.

**Table 1:** Demographic and clinical characteristics of study subjects.

	Elite controllers	ART+ treated	Viremic	Healthy controls
Patients, *n*	19	19	8	17
Age, years, median (range)	46 (28–68)	52 (40–68)	33 (24–71)	49 (27–65)
Male, *n* (%)	10 (53)	16 (84)	4 (50)	9 (53)
Ethnicity, *n* (%)				
Caucasian	8 (42)	14 (73.5)	5 (62.5)	16 (94)
Black	10 (53)	4 (21)	3 (37.5)	0 (0)
Other	1 (5)	1 (5.5)	0 (0)	1 (6)
Mode of transmission, *n* (%)				
Heterosexual	10 (53)	10 (52.5)	5 (62.5)	NA
MSM	4 (22)	5 (26)	2 (25)	NA
IVDU	2 (10.5)	3 (16)	0 (0)	NA
Blood products	2 (10.5)	1 (5.5)	0 (0)	NA
Unknown	1 (5)	0 (0)	1 (12.5)	NA
Years since diagnosis, median (min–max)	9 (2.7–32.8)	20 (13–31)	0 (0–1.5)	NA
HIV subtype, *n* (%)				
B	2 (10.5)	2 (10.5)	4 (50)	NA
C	5 (26)	1 (5.5)	1 (12.5)	NA
CRF	2 (10.5)	1 (5.5)	2 (25)	NA
Other or ND	10 (53)	15 (78.5)	1 (12.5)	NA
Clinical parameters at time of sampling, median (min–max)				
CD4+ T-cell count (cells/μl)	950 (480–1,655)	550 (360–1,160)	418.5 (157–700)	NA
CD4+ T-cell %	46 (21–60)	34 (21–57)	27.5 (7–43)	NA
CD8+ T-cell count (cells/μl)	780 (505–2,055)	580 (230–1,270)	823.5 (300–1,572)	NA
CD8+ T-cell %	32.5 (22–64)	39 (19–55)	47 (31–68.5)	NA
CD4+/CD8+ ratio	1.475 (0.33–2.79)	0.9 (04–3)	1.475 (0.33–2.79)	NA
HIV RNA (copies/ml)	<19 (19–225)	<19 (19–19)	7,697 (1,897–55,088)	NA

*MSM, men who have sex with men; IVDU, intravenous drug use; ND, not determined; NA, not available*.

The Regional Ethical Council (Stockholm, Sweden 2009/1485-31, 2013/1944-31/4, and 2014/920-32) approved the study. All participants provided written informed consent in accordance with the Declaration of Helsinki.

### Cell Preparation and Antibody Reagents

Peripheral blood mononuclear cells (PBMCs) were isolated from EDTA-treated whole blood by using Hypaque-Ficoll (GE Healthcare) density gradient centrifugation and then cryopreserved in fetal bovine serum (Sigma-Aldrich) containing 10% DMSO (Sigma-Aldrich). The flow cytometry panel was tested on all study subjects within 1-month time interval to avoid intra- and interindividual differences of the analysis. The following antibodies were used: anti-CD14 V500 (clone M5E2), anti-CD19 V500 (clone HIB19), anti-CD152 (CTLA-4) (clone BNI3), anti-Helios AF488 (clone 22F6), anti-HLA-DR BV605 (clone G46-6) (BD Bioscience); anti-CD3 BV570 (clone UCHT1), anti-CD8 BV711 (clone RPA-T8), anti-CD25 PE-Cy5 (clone BC96), anti-CD38 A700 (clone HIT2), anti-CD27 BV785 (clone O323), anti-CCR7 APC-Cy7 (clone G043H7), and anti-PD-1 BV421 (clone EH12.2H7) (BioLegend); anti-CD45RO ECD (clone UCHL1) (Beckman Coulter); anti-CD4 PE-Cy5.5 (clone SK3), anti-FoxP3 PE (clone PCH101), and anti-Tigit PE-Cy7 (clone MBSA43) (eBioscience). LIVE/DEAD Aqua amine dye (Life Technologies) was used to discriminate dead cells and debris.

### Antibody Staining

Peripheral blood mononuclear cells were thawed and washed twice in R10 [RPMI-1640 medium, AQMEDIA (Sigma-Aldrich) containing 10% FBS, 50 IU/ml penicillin, 50 µg/ml streptomycin, 10 mM HEPES (Sigma-Aldrich), and 10 U/ml DNase I (Roche Diagnostics)]. After final wash, the cells were resuspended in R10 to a final concentration of 1 × 10^6^ cells/ml and then rested for 2 h at 37°C.

The PBMCs were next transferred to 5 ml round-bottom Falcon FACS tubes (Corning) and washed twice in PBS containing 2 mM EDTA before incubated at 37°C for 10 min with anti-CCR7 antibody. The cells were then further incubated for 30 min at room temperature in the dark with a LIVE/DEAD Aqua amine dye containing the remaining extracellular antibodies. After staining, the cells were washed in PBS:EDTA, fixed and permeabilized using the FoxP3 transcription factor buffer kit (eBioscience) before further incubated with monoclonal antibodies against CD3, FoxP3, Helios, and CTLA-4 for 1 h in the dark. The cells were then washed twice with Perm Wash solution and resuspended in PBS containing 1% paraformaldehyde. All specimens were acquired on the flow cytometer within 2 h after fixation.

### Flow Cytometry Analysis and Statistical Analysis

Peripheral blood mononuclear cells were analyzed on a 4-laser LSR Fortessa (BD Bioscience). Approximately 1,500,000 events were recorded per specimen. In addition, antibody capture beads (BD Bioscience) were used for compensation and prepared individually by separate staining of all the antibodies used in the experiment. FlowJo X 10.0.7r2 (Treestar) was used for gating analysis, and statistical analysis was performed with GraphPad Prism 6.0. Analysis and graphical representation of cell surface inhibitory markers were conducted using data analysis program SPICE (version 5.35001) (28).

## Results

### HIV-1 ELCs Demonstrate Low Co-Expression of Inhibitory Receptors on Memory CD4+ T Cells

T cell exhaustion is a hallmark of HIV-1 disease. Despite CD4+ T cells are critical for control of viral infections, less is known about their co-expression pattern of inhibitory receptor in HIV-1 infection and in relation to healthy subjects. Our preliminary studies showed negligible expression of Tim-3, LAG-3, and CD160 on CD4+ T cells and a strong association of 2B4 and KLRG-1 with effector CD4+ T cells (data not shown), similar to as described in other studies (15, 21, 29, 30). We therefore focused on assessing the expression pattern of PD-1, CTLA-4, and TIGIT on different CD4+ memory T cell populations, defined by surface markers CD27, CD45RO, and CCR7, using multiparametric flow cytometry. Representative gating schemes for the flow cytometry analysis of the individual inhibitory molecule expression with respect to the different CD4+ maturation states (29) are presented in Figure S1 in Supplementary Material. Naive CD4+ T cells, which contain the lowest expression level of inhibitory molecules, were used as reference to define cells that had positive expression of respective markers.

**Figure 1:**
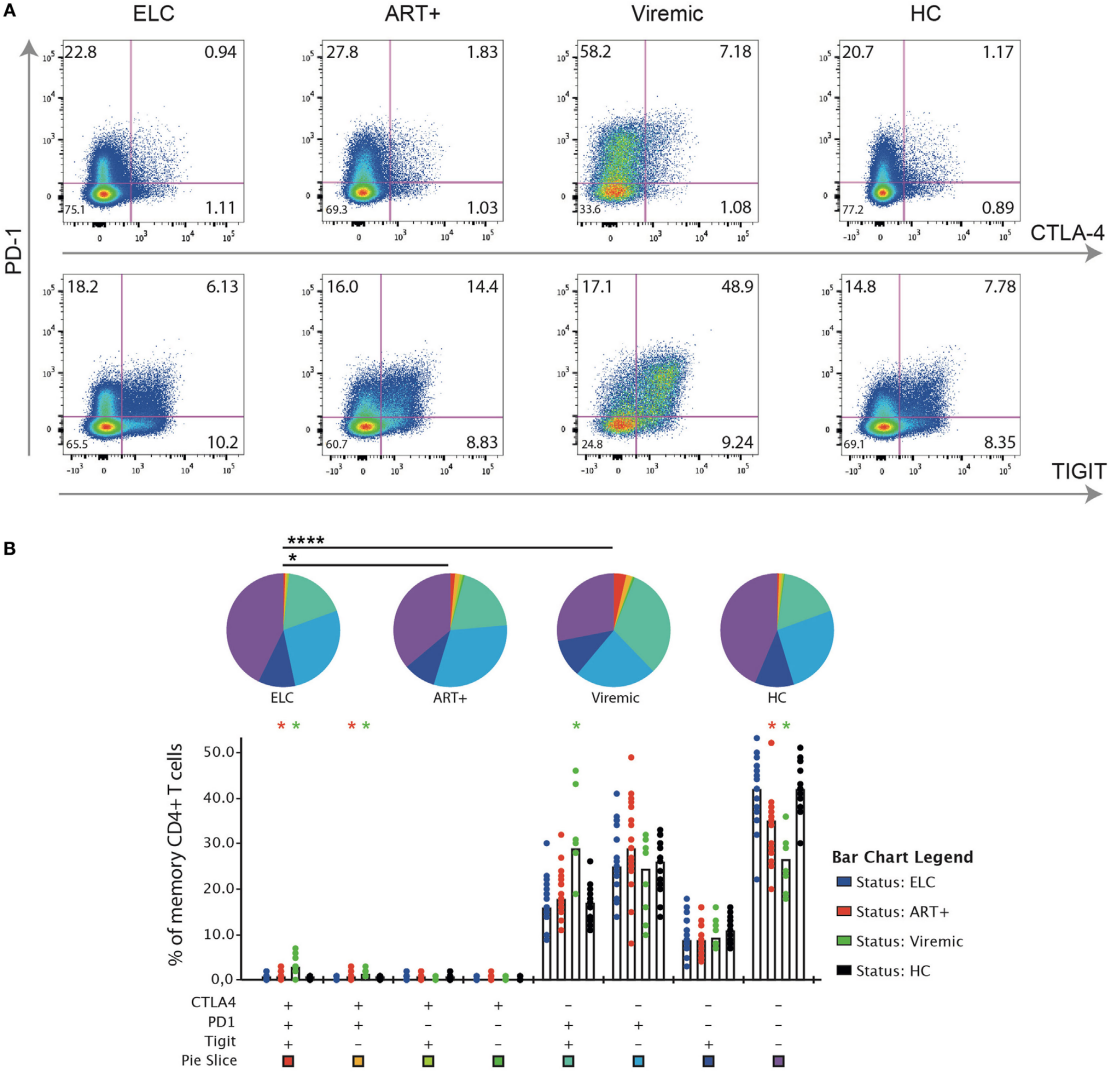
Expression patterns of inhibitory receptors on CD4+ T cell in elite controllers are significant different to cART+ and viremic patients but similar to healthy individuals. **(A)** Gating strategy illustrating the identification of cells positive for programmed cell death-1 (PD-1), CTLA-4, and TIGIT, respectively, on memory CD4+ T cell. Representative plots for an elite controller (ELC), long-term cART-treated subject (cART+), viremic untreated subject (viremic), and uninfected healthy individual [healthy control (HC) group]. **(B)** SPICE analysis of different combinations of PD-1, CTLA-4, and TIGIT on memory CD4+ T cells among the different patient groups. Bars represent the median within each group. Wilcoxon matched-pairs single rank test were performed to compare difference of expression between ELCs and other patient groups (*p ≤ 0.05), and permutation test was performed between pie charts (*p ≤ 0.05 and ****p ≤ 0.0001).

The expression of PD-1, CTLA-4, and TIGIT on bulk memory CD4+ T cells differed between ELCs and long-term cART and viremic subjects, respectively (Figures 1A,B). The frequencies of single expressing PD-1+, CTLA-4+, and TIGIT+ CD4+ T cells were significantly lower in ELCs compared with viremic subjects (Figure S2 in Supplementary Material). Although the single expression of PD-1, CTLA-4, and TIGIT on CD4+ T cells was not significantly different between ELCs and cART+ subjects, the expression was showing a trend toward statistical significance (Figure S2 in Supplementary Material). Further analysis revealed that co-expression of multiple inhibitory receptors was significantly different between the patient groups to a higher degree than single marker expression (Figure 1B). For example, CD4+ T cells co-expressing PD-1, CTLA-4, and TIGIT were significantly lower in ELCs compared with both long-term cART (*p* = 0.03) and viremics (*p* = 0.002). Also, CD4+ T cells expressing PD-1 and CTLA-4, but not TIGIT, were significantly lower in ELCs compared with long-term cART subjects (*p* = 0.003) and viremics (*p* = 0.001). On the contrary, CD4+ T cells negative for all makers were higher in ELCs than long-term cART and viremics (Figure 1B). The inhibitory receptor expression pattern (summarized in pie chart form) in ELCs was significantly different to cART+ (*p* = 0.04) and viremics (*p* < 0.001), but not to healthy individuals. Neither the expression pattern (pie charts) nor the different combinations of marker expression showed any difference between ELCs and healthy individuals, suggesting that the state of CD4+ T cell exhaustion in ELCs is equivalent to healthy subjects.

### Expression Patterns of Inhibitory Receptors Vary across Distinct Memory CD4+ T Cell Subsets

**Figure 2:**
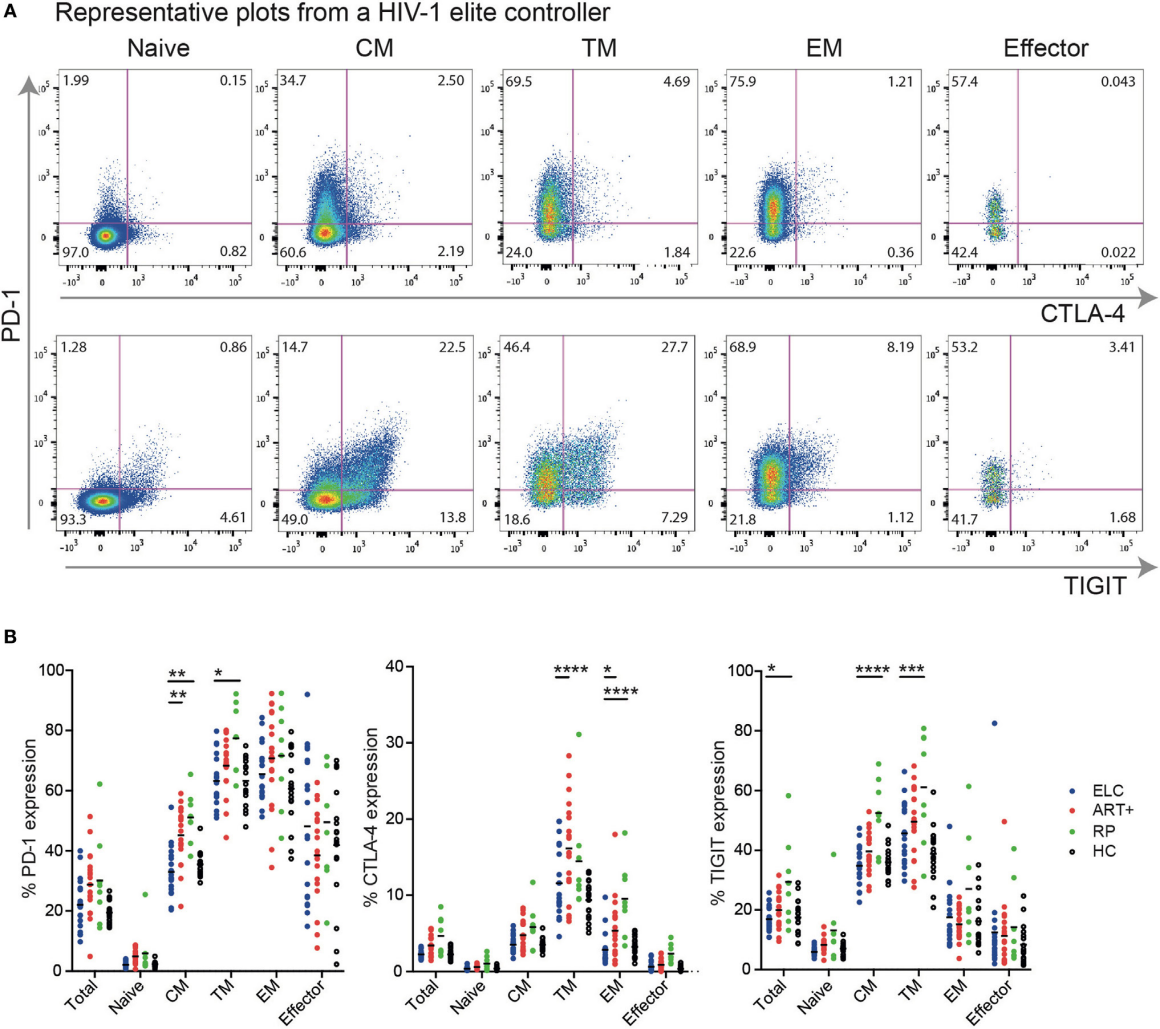
Expression patterns of inhibitory receptors vary across distinct memory CD4+ T cell subsets. **(A)** Gating strategy illustrating the identification of cells positive for programmed cell death-1 (PD-1), CTLA-4, and TIGIT, respectively, on CD4+ T cell subsets; naive T cells (Naive), central memory T cells (CM), transitional memory T cells (TM), effector memory T cells (EM), and effector T cells (Effector). Representative plots for an elite controller. **(B)** Data showing the percentage of different CD4+ T cell memory subsets expressing PD-1, CTLA-4, and TIGIT, respectively, within the total CD4+ T cell pool, from elite controllers (ELC, *n* = 19, blue dots), cART-treated subjects (cART+, *n* = 19, red dots), untreated chronically infected (viremic, *n* = 8, green dots), and healthy subjects [healthy control (HC), *n* = 17, black dots]. Horizontal lines indicate median value. *p* Values were calculated using two-way ANOVA with Bonferroni correction. *p ≤ 0.05, **p ≤ 0.01, ***p ≤ 0.001, and ****p ≤ 0.0001.

Next, we investigated the expression levels of each inhibitory molecule on distinct memory CD4+ T cell subsets. Representative gating schemes for the flow cytometry analysis of single inhibitory molecule expression on different CD4+ cell subsets are presented in Figure 2A. We found that the expression of PD-1 and TIGIT was in general higher on all cell subsets compared with CTLA-4 (Figure 2B). PD-1 was expressed to some degree on all memory CD4+ T cell subsets, with transitional memory (TM; CCR7− CD27+ CD45RO+) and effector memory (EM; CCR7− CD27− CD45RO+) displaying the highest expression. Similar memory expression pattern was also observed for CTLA-4 and TIGIT. Effector cells (CCR7− CD27− CD45RO−) expressed some PD-1 and TIGIT, but generally very low levels of CTLA-4. Among TM cells, ELCs displayed a significant lower expression of PD-1 (*p* < 0.05) and TIGIT (*p* < 0.001) compared with viremics and lower expression of CTLA-4 compared with cART+ (*p* < 0.0001). PD-1 expression on CM in ELCs was significantly lower compared with both cART+ and viremics. The mean fluorescence intensity (MFI) of PD-1, CTLA-4, and TIGIT on different memory subset showed primarily differences between ELCs and viremics (Figure S3 in Supplementary Material). ELCs did not display a significant difference to HC in regards to any of the markers.

### Increased Co-Expression of Inhibitory Receptors Correlates with T Cell Activation

**Figure 3:**
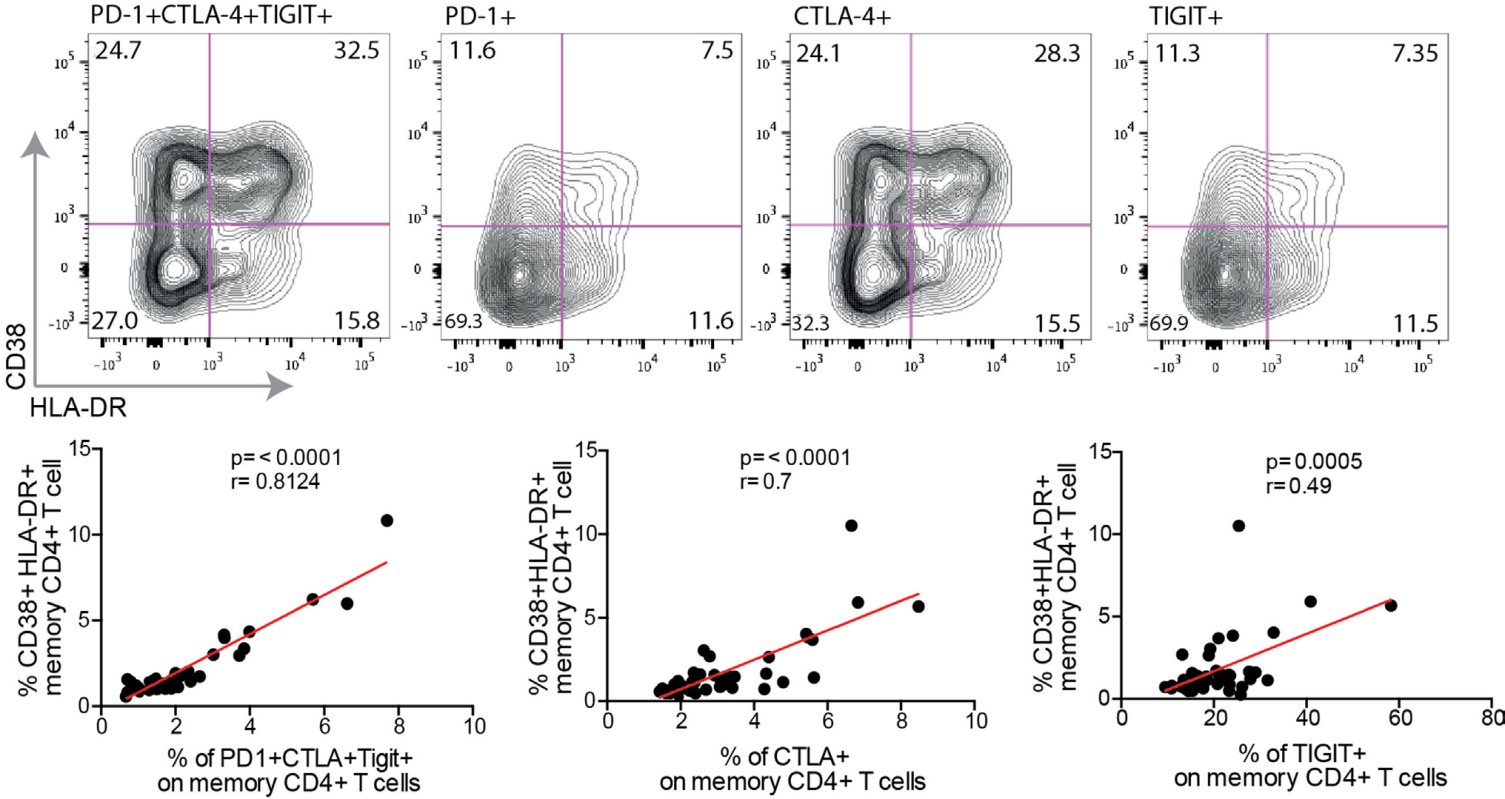
Co-expression of inhibitory markers correlate to T cell activation. The surface phenotype of activated T cells from a single HIV+ individual is shown in exhausted cell populations [programmed cell death-1 (PD-1)+ CTLA-4+ TIGIT+, PD-1+, CTLA-4+, or TIGIT+], black contour plot. Spearman nonparametric test was used for correlations analyses (*n* = 46). Lines indicate correlations detected by Prism software version 6.0.

We next sought to determine if increased expression levels of PD-1, CTLA-4, and TIGIT were associated with the immune activation levels. Although some of the PD-1+, CTLA-4+, and TIGIT+ CD4+ T cells expressed the immune activation markers CD38 and HLA-DR, many of these exhausted cells were CD38− HLA-DR− (Figure 3). Nevertheless, a strong correlation was found between PD-1+ CTLA-4+ TIGIT+ CD4+ T cells, and immune activation, both single and dual CD38 and HLA-DR expressing cells (*p* < 0.0001 for all analysis). Particularly, increased single expression of CTLA-4 was strongly associated with single expression of CD38 (*r* = 0.46, *p* = 0.001) and HLA-DR (*r* = 0.65, *p* < 0.0001), and dual expressing cells (*r* = 0.7, *p* < 0.0001) (Figure 3), suggestive of a clear link between CTLA-4 and immune activation. Surprisingly, no correlation was found between the activation status and PD-1 expression. The expression of TIGIT was significantly correlated to cells expressing both CD38 and HLA-DR (*r* = 0.49, *p* < 0.001), but not significantly associated to single expression of CD38 and HLA-DR (Figure 3).

### Inhibitory Receptor Expression Is Linked to Higher Expression Levels of Helios and FoxP3

**Figure 4:**
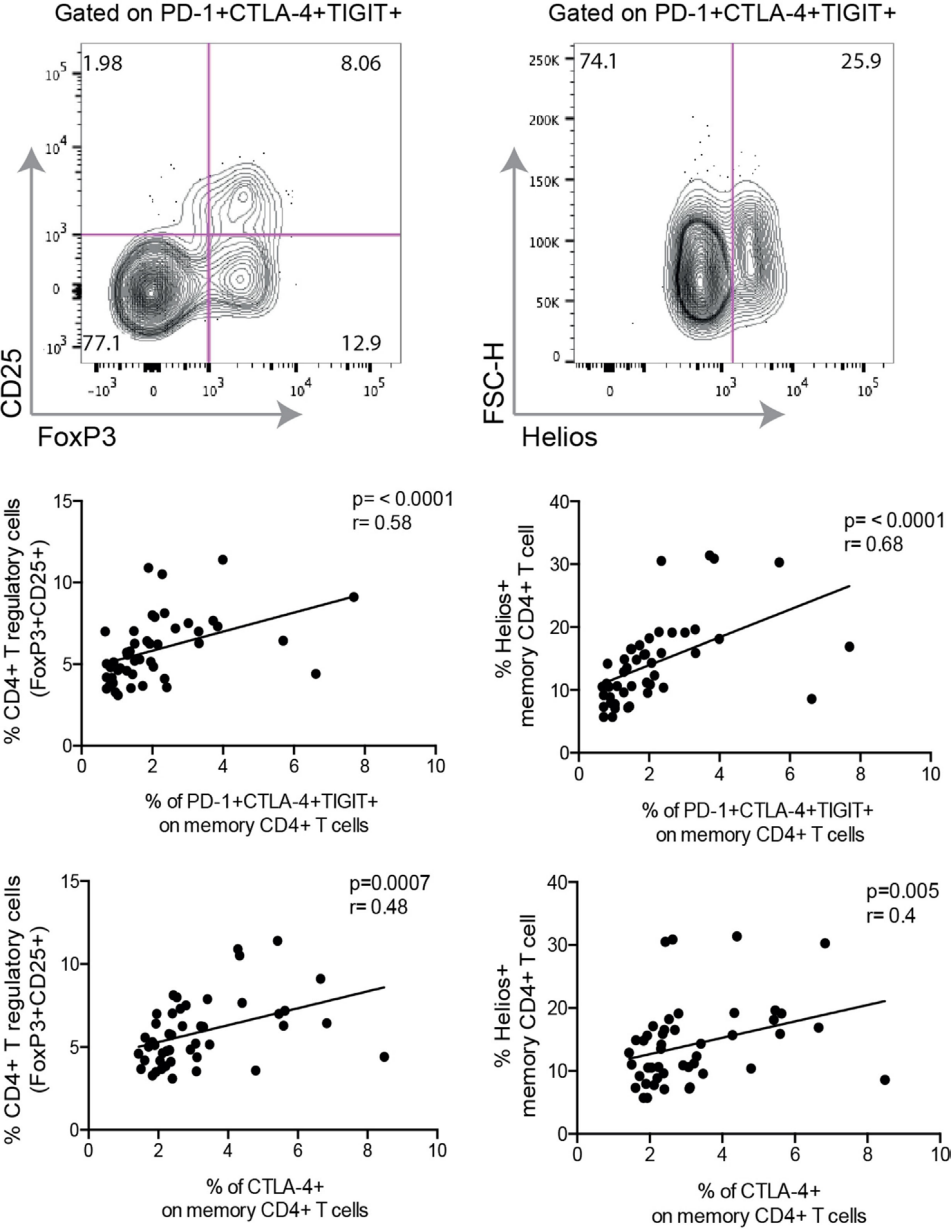
Expression of inhibitory molecules correlate to frequency of T-regulatory cells and the expression of transcription factor Helios. Representative FACS plot from a single HIV+ individual displaying exhausted T cells [programmed cell death-1 (PD-1)+ CTLA-4+ TIGIT+] expressing markers of regulatory T cells (CD25 and FoxP3) and Helios. Spearman nonparametric test was used for correlations analyses (*n* = 46). Lines indicate correlations detected by Prism software version 6.0.

Studies have revealed that T cell exhaustion is connected to a complex network of transcription factors (18–20). Crawford et al. demonstrated an association between exhausted CD4+ T cells and the transcription factors Ikzf2 (Helios) and FoxP3 (20); two transcription factors highly expressed in T-regulatory cells (Tregs). In our cohort, we found a strong correlation between PD-1+ CTLA-4+ TIGIT+ CD4+ T cells and the frequency of CD4+ FoxP3+ CD25+ T cells (*r* = 0.58, *p* < 0.001) (Figure 4). A positive correlation was also found for CTLA-4 (*r* = 0.48, *p* < 0.001) and TIGIT expressing cells (*r* = 0.42, *p* = 0.003), respectively. Despite the positive correlation between exhausted CD4+ T cells and the frequency of FoxP3+ CD25+ CD4+ T cells, we could observe that not all exhausted cells had this phenotype, based on the FACS plot in Figure 4. The same correlation pattern was also observed with the expression of Helios. Helios-expressing CD4+ cells were positively correlated to cells co-expressing all three inhibitory receptors (*r* = 0.68, *p* < 0.001), as well as cells expressing CTLA-4 (*r* = 0.4, *p* = 0.005). We found no correlation between Helios and TIGIT, and PD-1 was not correlated to either expression of Helios or frequency of FoxP3+ CD25+ CD4+ T cells.

### Clinical Parameters of HIV-1 Disease Progression Are Associated with CD4+ T Cell Exhaustion

**Figure 5:**
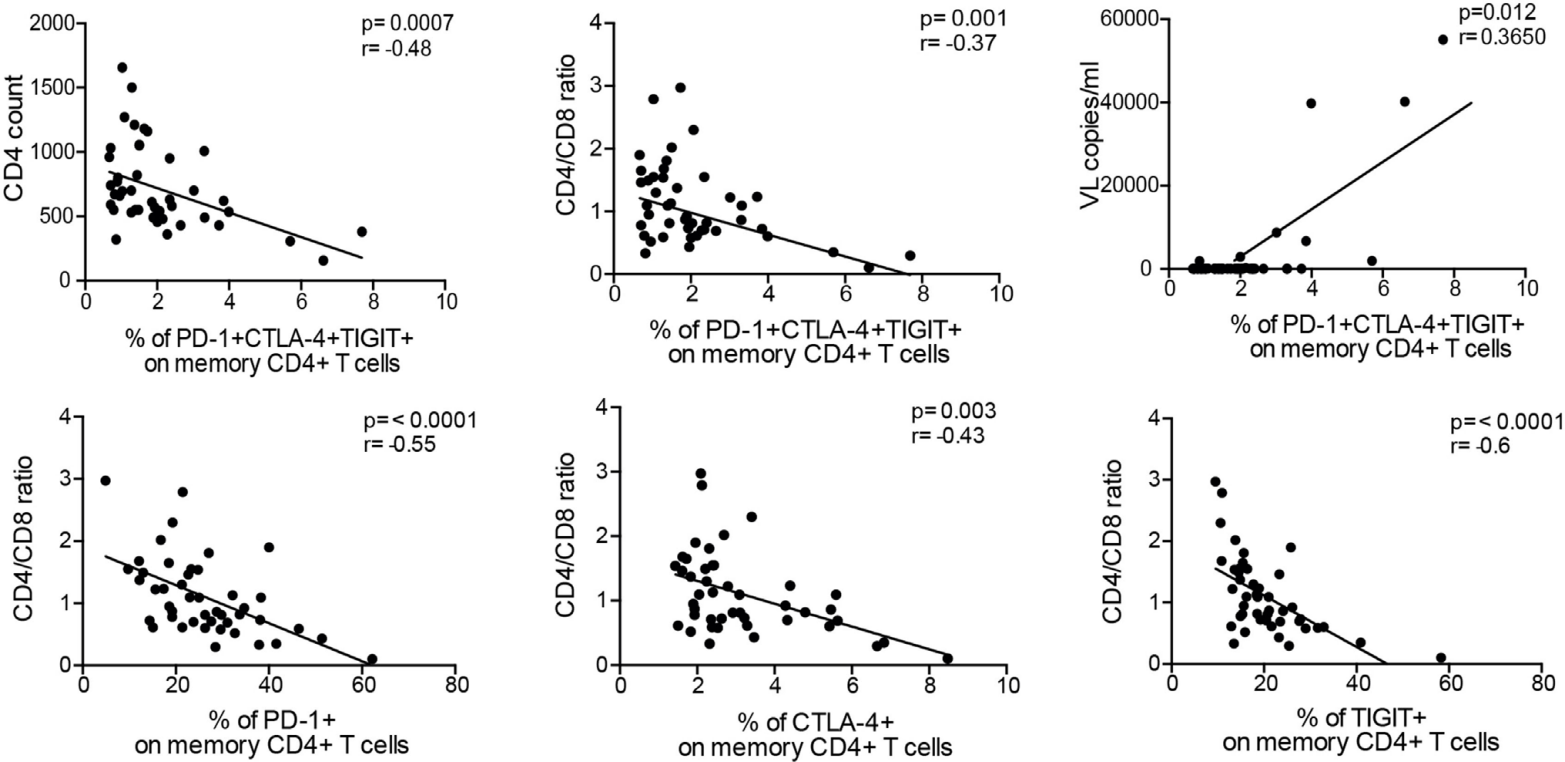
Exhausted CD4+ T cells correlate to viremia and clinical parameters. Percentage of co-expression of inhibitory molecules on memory CD4+ T cells correlates to CD4 count, CD4/CD8 ratio, and plasma viral load. Of all clinical parameters, the CD4/CD8 ratio was the preeminent parameter displaying the strongest correlations to single expression of the inhibitory molecules as well as co-expression. Spearman nonparametric test was used for correlations analyses (*n* = 46). Lines indicate correlations detected by Prism software version 6.0.

We finally investigated the relationship between T cell exhaustion and routine laboratory parameters (CD4 count, viral load, and CD4/CD8 ratio). We observed an inverse correlation between CD4+ T cells co-expressing all inhibitory receptors to several parameters: CD4 count (*r* = −0.48, *p* < 0.001), CD4/CD8 ratio (*r* = −0.37, *p* = 0.001), and viral load (*r* = 0.37, *p* = 0.012) (Figure 5). Particularly, the CD4/CD8 ratio displayed significant correlations to single expression of all inhibitory molecules. This confirms data from our previous studies demonstrating that the CD4/CD8 ratio is a suitable predictor of T cell dysfunction (21, 31). T cell exhaustion and clinical parameters were independent of the racial distribution within the ELC group (Figure S4 in Supplementary Material) and patient’s age (data not shown).

## Discussion

Increased expression of inhibitory receptors on T cells is linked to poor control of chronic viral infections and cancer, and the blockade of these receptors has revolutionized the treatment agenda in the field of multiple cancers (16, 17). However, most studies to date have focused on studying exhaustion of CD8+ T cells, and to a less degree what role this process plays for CD4+ T cells in HIV-1 infection. In this study, we demonstrate that HIV-1 ELCs maintain low expression levels of cell markers associated with T-cell exhaustion than viremic and aviremic HIV-infected subjects on cART. We furthermore demonstrate that the frequency of inhibitory receptor expression on CD4+ T cells is highly associated with immune activation, regulatory transcription factors, and markers of HIV-1 disease progression. Overall, our data suggest that the accumulation of inhibitory receptors on CD4+ T cells is increased in HIV-infected subjects also on long-term cART, but not in subjects naturally controlling the infection.

Several studies have demonstrated that T cell exhaustion is a hallmark of HIV-1 infection. While previous studies have assessed exhausted CD8+ T cells in the context of ELCs, few studies to date have assessed the co-expression pattern of inhibitory receptors on CD4+ T cells in HIV-infected ELCs. Our data demonstrate that the pattern of inhibitory receptor expression, defined by expression of PD-1, CTLA-4 and TIGIT on memory CD4+ T cells, was significantly lower in ELCs compared with well-treated and viremic subjects and similar to the levels in healthy subjects. The low expression levels seen in the ELCs are a bit surprising, given that other studies have previously demonstrated that ELCs have elevated T cell activation associated with progressive disease (32, 33). However, these studies have mostly been conducted on CD8+ T cells, and we recently have described that multiple inhibitory receptors are indeed higher on CD8+ T cells in ELCs (34). In line with our results, another study has observed low PD-1 MFI levels on CD4+ T cells in ELCs that was similar to HIV-seronegative individuals (35), indicating that inhibitory receptor levels are not associated with excessive immune activation in ELCs. Our cohort consists of individuals with stable, non-declining CD4 cell counts, generally with undetectable virus levels over a course for several years. Given that the definition of ELCs can vary somewhat between different cohorts (3), our results might be different than cohorts including a larger proportion of ELCs with disease progression.

Previous studies on CD8+ T cells have found that expression of multiple inhibitory receptors cooperates to limit T cell activation and is associated with an exhausted phenotype (7). Our analysis on CD4+ T cells in the context of HIV infection are in line with these findings and suggest that single expression of all inhibitory receptors did not generate considerable differences between the patient groups. However, when investigating their co-expression pattern, we found significant differences between all HIV-infected groups. The frequency of cells co-expressing PD-1, CTLA-4, and TIGIT was lower in ELCs in comparison with well-treated and viremic subjects, and the same difference was observed with the frequency of cells expressing PD-1 and CTLA-4. These data indicate that solely looking at the single expression of exhaustion-associated markers is not sufficient to determine the state of exhausted T cells. Rather, proper evaluation must focus on investigating cells co-expressing several inhibitory markers.

Elevated chronic immune activation is a hallmark of HIV disease (36). Similar to previous studies, our results demonstrate a strong association between the level of inhibitory receptors and immune activation markers on CD4+ T cells. Despite the strong positive association, our analysis revealed that only a fraction of cells positive for PD-1, CTLA-4, and/or TIGIT expressed immune activation markers. This implicates that exhausted cells are not necessarily activated, yet there is a strong relationship present between the two factors. We also identified positive associations between the expression of inhibitory receptors and expression of the two transcription factors Helios and FoxP3, classically linked to Tregs. Recent work has demonstrated that conventional Tregs (CD25+ FoxP3+) are composed of many subpopulations, especially Tregs expressing Helios together with TIGIT possess high suppressive activity (37–39). Our data suggest that a higher degree of exhausted CD4+ T cells during HIV-1 infection is partly linked to higher frequency of CD25+ FoxP3+ CD4+ T cells. However, similar to what was observed with the association to immune activation, only a small fraction of exhausted CD4+ T cells expressed FoxP3 or Helios despite the strong correlation, indicating that other subpopulations of conventional CD4+ T cells exist expressing high levels of inhibitory receptors. Finally, when investigating the relationship between T cell exhaustion and clinical parameters (CD4 count, VL, etc.), there was a clear association with disease progression as VL increased and CD4 count decreased with elevated expression of inhibitory receptors. Similar to our previous observations (21, 31, 40), the CD4/CD8 ratio was the clinical parameter displaying the strongest correlation to all three inhibitory markers on CD4+ T cells.

This study has some limitations. The racial distribution and number of ELC patient samples might affect the power of statistical analysis but this is, however, beyond our control as the prevalence of ELCs among the HIV-infected community is low and somewhat dependent on the definition of an ELC (3). Although we acknowledge the lack of functional data in our study and its importance, we do present here an observational study in a unique group of HIV-1-infected subjects that identifies the state of exhausted cells within the CD4+ T cell repertoire and its proper correlation to routine laboratory markers. Indeed, a recent study demonstrated that blocking specific inhibitory receptors lead to differential expression patterns of certain cytokines in CD4+ T cells, suggesting that unique functional properties can be restored by blocking different checkpoint inhibitors (22).

Overall, our data reveal that ELCs are able to maintain low expression of several inhibitory receptors on CD4+ T cells, despite ongoing viral infection for a prolonged period of time. We confirm that ELCs harbor a “healthy state” of distinct CD4+ memory T cell subsets and a relationship to disease progression parameters that together might play part in maintenance of their control status. Furthermore, these results implicate that low CD4+ T cell exhaustion is an important component for effective control of HIV-1.

## Ethics Statement

The Regional Ethical Council (Stockholm, Sweden 2009/1485-31, 2013/1944-31/4, and 2014/920-32) approved the study. All participants provided written informed consent in accordance with the Declaration of Helsinki.

## Author Contributions

KN, MRB, AS, and MB conceived and designed the experiments. KN and AS coordinated samples collection. KN, SN, and MB prepared the samples, performed the experiments, analyzed the data, and wrote the paper. All the authors reviewed and/or edited the manuscript.

## Conflict of Interest Statement

The authors declare that the research was conducted in the absence of any commercial or financial relationships that could be construed as a potential conflict of interest.

## Supplementary Material

Figure S1 Gating strategy for different memory CD4+ T cell populations. Representative gating scheme for determination of different memory CD4+ T cell subsets is shown for a non-infected healthy subject. Gates were based on the expression of CD27, CD45RO, and CCR7. Naive: CD27+ CD45RO− CCR7+; central memory T cells (CM): CD27+ CD45RO+ CCR7+; transitional memory T cells (TM): CD27+ CD45RO+ CCR7−; effector memory T cells (EM): CD27-CD45RO+ CCR7−; and effector T cells (Effector): CD27− CD45RO− CCR7−.

Figure S2 **(A)** Frequency and **(B)** mean fluorescence intensity (MFI) of programmed cell death-1 (PD-1), CTLA-4, and TIGIT on memory CD4+ T cells in HIV-seronegative and HIV-seropositive subjects. Horizontal lines indicate median with interquartile range. *p* Values were calculated using Kruskal–Wallis test. **p* ≤ 0.05, ***p* ≤ 0.01, and ****p* ≤ 0.001.

Figure S3 Data showing different CD4+ T cell memory subsets expressing mean fluorescence intensity (MFI) of programmed cell death-1 (PD-1), CTLA-4, and TIGIT, respectively, within the total CD4+ T cell pool, from elite controllers (blue dots), ART-treated subjects (red dots), untreated chronically infected (green dots), and healthy subjects (black dots). Horizontal lines indicate median value. *p* Values were calculated using two-way ANOVA with Bonferroni correction. **p* ≤ 0.05, ***p* ≤ 0.01, ****p* ≤ 0.001, and *****p* ≤ 0.0001.

Figure S4 Data showing the inhibitory receptor distribution, CD4 count, and CD4/CD8 ratio between Blacks (African origin) and Others (Caucasians and Latins). Horizontal lines indicate median with interquartile range. *p* Values were calculated with either Mann–Whitney or Kruskal–Wallis test.
