# Supplementary figures and images for: Human Immunodeficiency Virus Type-1 Elite Controllers Maintain Low Co-Expression of Inhibitory Receptors on CD4+ T Cells
Source: Front Immunol. 2018 Jan 22;9:19. doi: 10.3389/fimmu.2018.00019 (PMC5786543; doi:10.3389/fimmu.2018.00019)

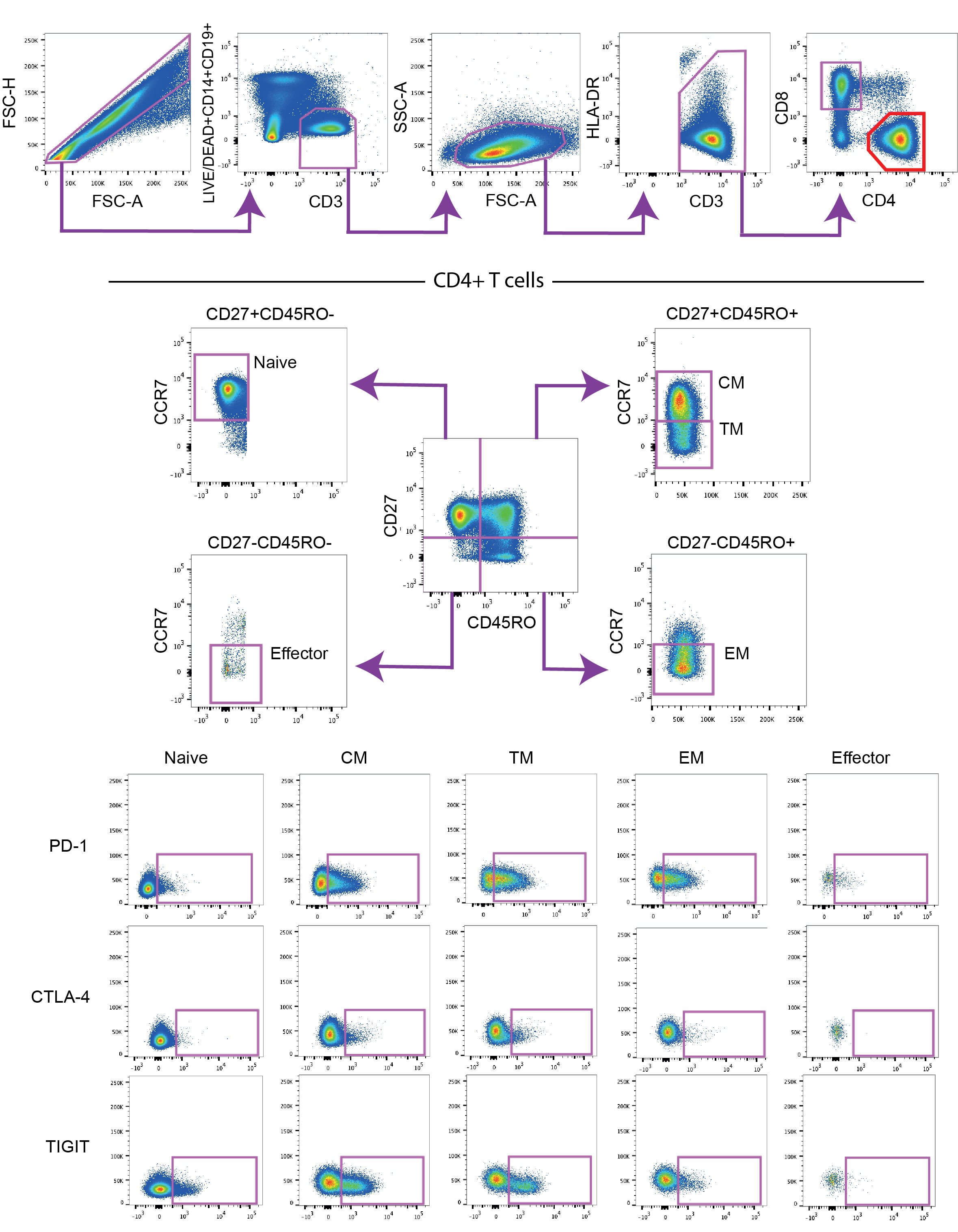

Supplement: Figure S1 — Gating strategy for different memory CD4+ T cell populations. Representative gating scheme for determination of different memory CD4+ T cell subsets is shown for a non-infected healthy subject. Gates were based on the expression of CD27, CD45RO, and CCR7. Naive: CD27+ CD45RO− CCR7+; central memory T cells (CM): CD27+ CD45RO+ CCR7+; transitional memory T cells (TM): CD27+ CD45RO+ CCR7−; effector memory T cells (EM): CD27-CD45RO+ CCR7−; and effector T cells (Effector): CD27− CD45RO− CCR7−. [file Image_1.JPEG]

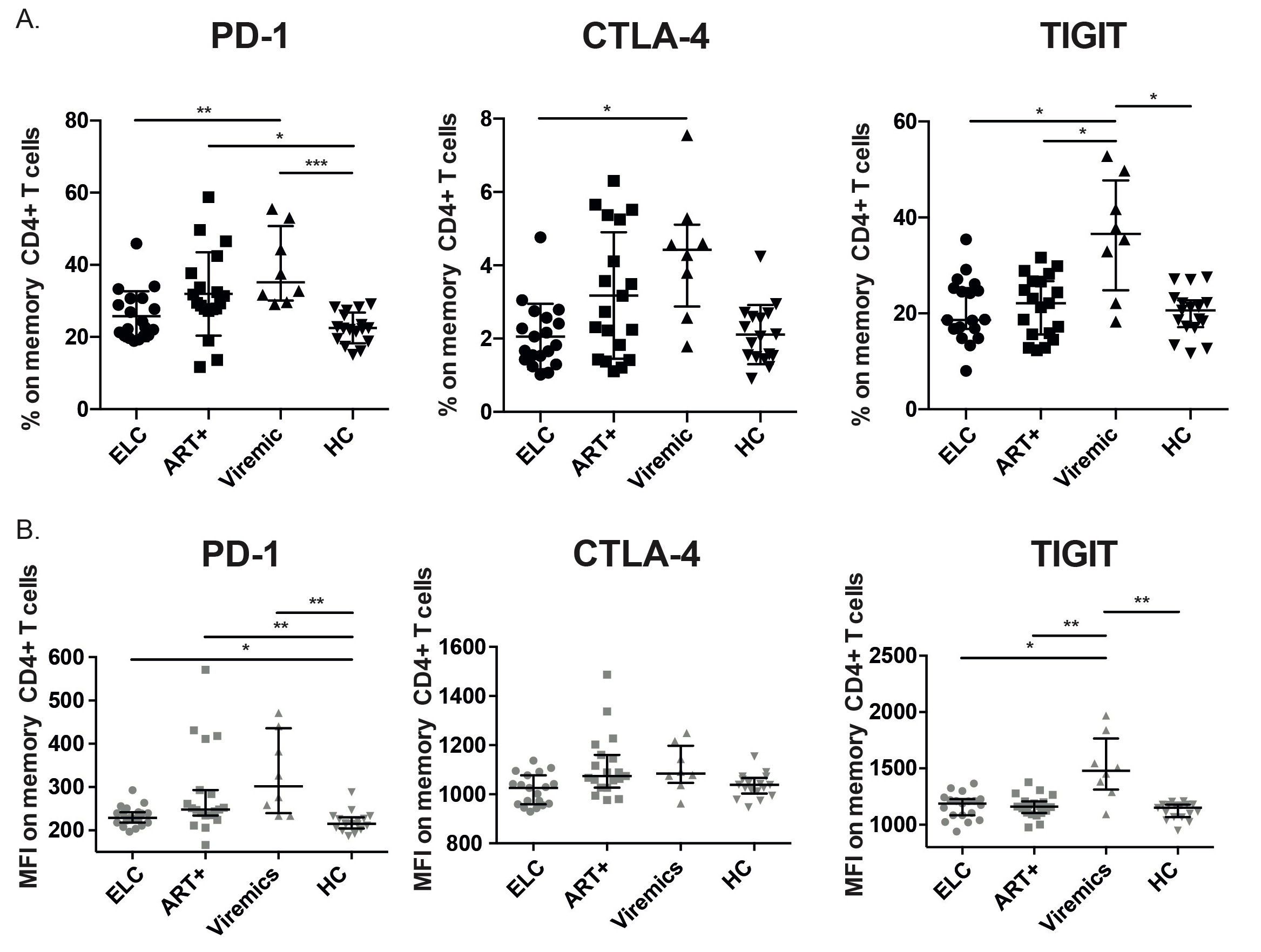

Supplement: Figure S2 — (A) Frequency and (B) mean fluorescence intensity (MFI) of programmed cell death-1 (PD-1), CTLA-4, and TIGIT on memory CD4+ T cells in HIV-seronegative and HIV-seropositive subjects. Horizontal lines indicate median with interquartile range. p Values were calculated using Kruskal–Wallis test. *p ≤ 0.05, **p ≤ 0.01, and ***p ≤ 0.001. [file Image_2.JPG]

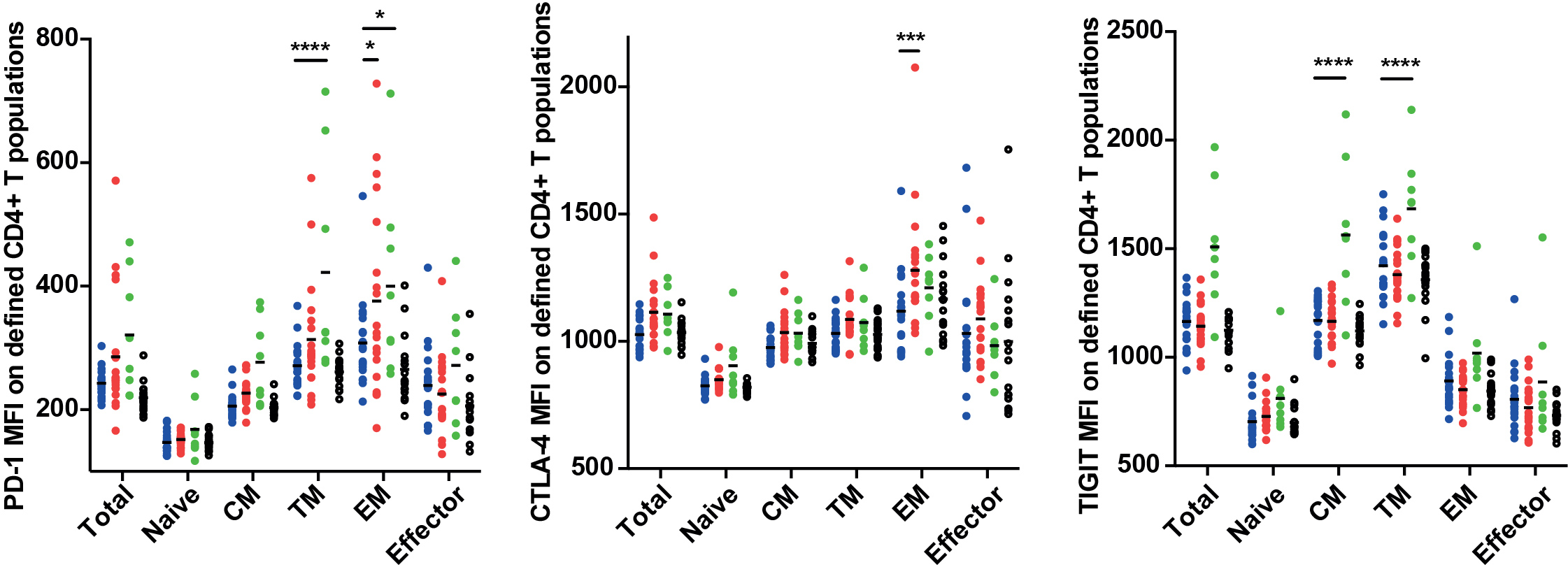

Supplement: Figure S3 — Data showing different CD4+ T cell memory subsets expressing mean fluorescence intensity (MFI) of programmed cell death-1 (PD-1), CTLA-4, and TIGIT, respectively, within the total CD4+ T cell pool, from elite controllers (blue dots), ART-treated subjects (red dots), untreated chronically infected (green dots), and healthy subjects (black dots). Horizontal lines indicate median value. p Values were calculated using two-way ANOVA with Bonferroni correction. *p ≤ 0.05, **p ≤ 0.01, ***p ≤ 0.001, and ****p ≤ 0.0001. [file Image_3.JPEG]

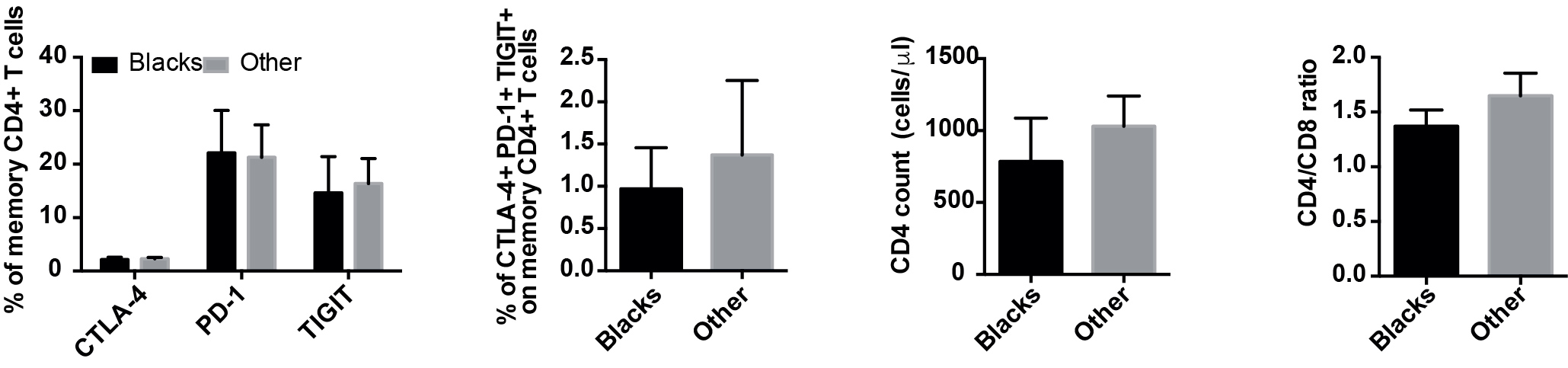

Supplement: Figure S4 — Data showing the inhibitory receptor distribution, CD4 count, and CD4/CD8 ratio between Blacks (African origin) and Others (Caucasians and Latins). Horizontal lines indicate median with interquartile range. p Values were calculated with either Mann–Whitney or Kruskal–Wallis test. [file Image_4.JPEG]
