# Supplementary material for: Human Immunodeficiency Virus Type-1 Elite Controllers Maintain Low Co-Expression of Inhibitory Receptors on CD4+ T Cells
Source: Front Immunol. 2018 Jan 22;9:19. doi: 10.3389/fimmu.2018.00019 (PMC5786543; doi:10.3389/fimmu.2018.00019)
Supplement: Supplementary file 5 [file Table_1.PDF]

Supplementary table 1. Characteristics of 19 patients in whom the plasma HIV RNA load has been undetectable in the absence of antiretroviral treatment.

Patient	Age	Gender	Ethnicity	Inclusion criteria*	Mode of Transmission	No. of years of HIV infection	HIV subtype	CD4 count, median (min-max)	CD4%, median (min-max)	CD8 count, median (min-max)	CD8%, median (min-max)	CD4/CD8 ratio, median (min-max)
#1	62	Male	Caucasian	2	Heterosexual	9	C	550 (380-610)	28 (21-30)	950 (660-1490)	48.5 (45-60)	0.59 (0.37-0.62)
#2	52	Male	Black	1	Heterosexual	3	C	955 (630-1180)	41 (33-46)	745 (100-860)	35 (34-39	1.18 (0.84-1.37)
#3	46	Female	Black	1	Heterosexual	14	B	653 (520-820)	42 (37.5-47)	490 (390-545)	32.5 (30.5-35)	1.32 (1.18-1.49)
#4	41	Female	Black	1	Heterosexual	5	C	765 (630-980)	31 (28.5-33)	1068 (900-1180)	42.3 (40.5-47)	0.72 (0.65-0.83)
#5	40	Female	Black	1	Heterosexual	3	*ND*	755 (660-790)	47 (45-47.5)	528 (480-610)	33.5 (32.5-35)	1.41 (1.3-1.46)
#6	44	Female	Black	1	Heterosexual	19	C	645 (508-880)	32 (27-38)	855 (730-1010)	47 (41-52)	0.65 (0.59-0.92)
#7	51	Male	Caucasian	1	MSM	11	*ND*	1258 (950-1354)	47 (42-50)	843 (610-986)	32 (30.5-35)	1.52 (1.22-1.59)
#8	56	Male	Caucasian	1	IVDU	29	*ND*	730 (400-1130)	39 (23-48)	559 (305-1025)	28.5 (21-40)	1.37 (0.68-1.9)
#9	68	Male	Caucasian	1	MSM	6	CRF 01 AE	1040 (801-1210)	46 (43-48)	657 (570-680)	28 (26-35)	1.68 (1.23-1.83)
#10	45	Male	Caucasian	1	Blood product	33	*ND*	714 (590-962)	36.5 (27.5-44)	649 (433-1352)	30 (21.7-56.5)	1.21 (0.49-1.73)
#11	49	Male	Black	2	Heterosexual	11	*ND*	673 (520-810)	21 (19-22)	2103 (1410-2450)	64 (60-65)	0.33 (0.3-0.36)
#12	55	Male	Caucasian	2	MSM	29	B	930 (778-1268)	43 (34-47)	621 (498-888)	27.3 (24-34)	1.49 (1.02-1.96)
#13	32	Female	Black	1	Blood product	8	*ND*	959 (777-1240)	43.3 (38-47)	610 (610-610)	30 (30-30)	1.32 (1.32-1.32)
#14	48	Male	Latin	1	MSM	4	*ND*	995 (870-1270)	39.5 (37-45.5)	885 (790-965)	34.3 (34-35)	1.1 (1.1-1.3)
#15	28	Female	Black	1	Unknown	5	*ND*	947 (792-1096)	37 (33-40.5)	949 (810-1072)	34 (32.5-37.5)	1.07 (0.95-1.26)
#16	40	Female	Black	1	Heterosexual	5	*ND*	606 (504-695)	45.8 (43-48)	403 (367-439)	35.4 (35.3-35.5)	1.34 (1.1-1.55)
#17	43	Female	Caucasian	1	Heterosexual	14	C	1096 (636-1535)	45 (38-57.5)	827 (530-1058)	33 (28-38)	1.42 (1.05-2.31)
#18	49	Female	Caucasian	1	IVDU	6	CRF 01 AE	1334 (1095-1620)	45.8 (39.5-48-5)	1000 (765-1358)	32.5 (28-44)	1.44 (0.9-1.68)
#19	36	Male	Black	2	Heterosexual	12	*ND*	1320 (921-1810)	60 (54-62)	511 (430-610)	23 (20-26.5)	2.69 (1.05-3.22)

NOTE. MSM, men who have sex with men; IVDU, intravenous drug use; ND, not determined;

* HIV RNA levels <75 copies/ml on minimum 3 consecutive determinations, spanning over at least 12-months ART-free period with all previous determinations below 1000 copies/ml (**1**) or HIV positive status for ≥10 years with minimum 2 HIV RNA level determinations, ≥90% of all HIV RNA determinations below 400 copies/ml (**2**).
